# Insect Derived Lauric Acid as Promising Alternative Strategy to Antibiotics in the Antimicrobial Resistance Scenario

**DOI:** 10.3389/fmicb.2021.620798

**Published:** 2021-02-26

**Authors:** Luca Borrelli, Lorena Varriale, Ludovico Dipineto, Antonino Pace, Lucia F. Menna, Alessandro Fioretti

**Affiliations:** Department of Veterinary Medicine and Animal Productions, Università degli Studi di Napoli Federico II, Naples, Italy

**Keywords:** post-antibiotic epoch, *Hermetia illucens*, lauric acid, MCFAs, novel antimicrobial lipids

## Abstract

Antibiotic misuse is greatly contributing to an increase in antimicrobial resistance (AMR) in humans and animals. Natural and synthetic alternative strategies are being investigated in human and veterinary medicine, but little attention is paid to the antimicrobial effects of edible lipids, such as medium-chain fatty acids (MCFAs) and monoglycerides. Among MCFAs, lauric acid (LA) and its monoglyceride derivative, glycerol monolaurate (GML), exhibit the strongest antimicrobial activity. Coconut and palm kernel oils are considered the main sources of LA. On the other hand, some edible insects (e.g., *Hermetia illucens*) are gaining interest as novel feed ingredients, due to the high amount of LA they contain as well as their numerous bioactive components, which provide many additional benefits to animal health. Although the beneficial effect of both MCFAs and LA is gradually being recognized, their high content within insects and, consequently, their possible role as antimicrobials, has not been well-reported. This mini review focuses on the anti-infective effects of the insect-derived MCFAs LA and its derivatives. We emphasize the potential of insect lipids, compared to the other vegetable sources, in the current global scenario where a sustainable and circular economy is required. Finally, we critically discuss the use and the benefits of edible insects such as favorable options as feed and food from the perspective of animal and human nutrition.

## Tackling the Rise of Antimicrobial Resistance. Any Lipidic Alternatives?

A New York Times headline from 1945 reads “Penicillin’s finder assays its future; Sir Alexander Fleming observed that improved dosage method is needed to extend use.” Despite this early warning, today, antimicrobial resistance (AMR) represents a global-scale public threat, and the world is now on the cusp of a “post-antibiotic era.” The research community is investing in various drug discovery strategies to develop new antimicrobial drugs, as conventional drug therapies are becoming increasingly ineffective and limited (Farha and Brown, 2019; Schultz et al., 2020). Every year, 700,000 patients die worldwide due to AMR, but the number could easily and dramatically reach 10 million by 2050 (Ghosh et al., 2019). Antibiotic abuse both in humans and animals has greatly contributed to an increase in AMR and has also caused the accumulation of these compounds in the environment by selecting resistant microorganisms and turning the environment into an enormous reservoir for AMR genes (Roca et al., 2015). Moreover, antibiotics misuse in animal production and the EU ban on their use in feed (Regulation EC/1831/2003) has led to an increase in the incidence of livestock disease and economic damage (Dabbou et al., 2020).

**FIGURE 1:**
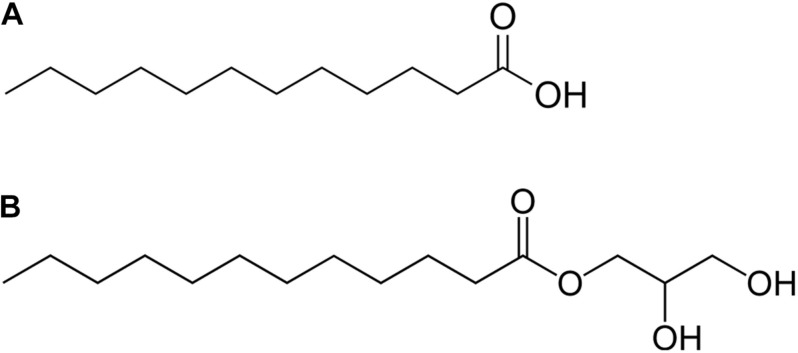
Chemical structure of lauric acid **(A)** and glycerol monolaurate **(B)**.

To date, numerous natural and synthetic alternative strategies are being investigated such as antibodies, bacteriophages, antimicrobial peptides, and alteration of the gut microbiota, predatory bacteria, or fecal transplant therapy (Kadouri et al., 2013; Aroniadis and Brandt, 2014; Mandal et al., 2014; Mahlapuu et al., 2016; Ghosh et al., 2019; Rello et al., 2019). Antimicrobial lipids, such as medium-chain fatty acids (MCFAs) and monoglycerides could also be a suitable alternative to antibiotics. MCFAs are originally an important component of the innate immune system in mammalian breast milk, skin, and mucosa and also induce host defense peptides expression in humans and animals (Zhou et al., 2019). Among MCFAs, lauric acid (LA) (Figure 1A) and its monoglyceride derivative, monolaurin (glycerol monolaurate, GML) (Figure 1B), exhibit the strongest antimicrobial activity. They can modulate intestinal health by regulating the level of IL-6 and TNF-α (Dabbou et al., 2020) and, not least, they are Generally Recognized As Safe (GRAS) by the United States Food and Drug Administration (Yoon et al., 2018). Their ability to destabilize the bacterial cell membrane makes them promising candidates among novel antimicrobials because these bacteria are also unlikely to acquire resistance to these compounds (Petschow et al., 1996; Schlievert and Peterson, 2012; Jackman et al., 2020).

Although the beneficial effect of MCFAs and LA is gradually being recognized, their content within insects has not been well-reported. Recently, insects have been receiving considerable attention as novel alternative feed ingredients because of their excellent nutritional properties and potential effects on animal health. They contain bioactive components, such as LA, antimicrobial peptides (defensins, cecropins, attacins, lebocins, lysozime proline-rich peptides, gloverins, and moricins) and the valuable biopolymer chitin, part of the exoskeletons of arthropods and chitosan, produced commercially by deacetylation of chitin which has antimicrobial, anti-tumor and immune-boosting properties (Sogari et al., 2019; da Silva Lucas et al., 2020; Moretta et al., 2020; Smets et al., 2020).

Among edible insects, *Hermetia illucens* is one of the main sources of LA (Spranghers et al., 2018). Thus, it may represent a good candidate given the growing market demand for edible insects as a new source of food, and also considering the need to find new strategies for antimicrobial resistance. Therefore, based on the studies presented so far, the perspectives for future applications of insect lipids might also be considered in human nutrition.

## Antimicrobial Effect of the Lauric Acid and Monolaurin

A recent overview reports the emerging antimicrobial properties of fatty acids (FAs) and their relation to virulence and quorum sensing (QS), such as diffusible signal factors (DSFs), acyl-homoserine lactones, and autoinducer-2 systems. The suppression of the expression of QS-regulated genes, especially those related to virulence (e.g., synthesis of toxins, fimbriae, hyphae, etc.) and other non-QS targets (proteins involved in efflux pumps, oxidative stress, and ergosterol synthesis) make FAs a new paradigm to cope with drug-resistant bacteria (Kumar et al., 2020). Of these, medium-chain fatty acids (MCFAs) and their monoglycerides have a broad spectrum of microbicidal activity against a wide range of pathogens both *in vitro* and *in vivo*, including multidrug-resistant bacteria, enveloped viruses, algae, fungi, and protozoa (Bergsson et al., 2001; Hilmarsson et al., 2007; Yoon et al., 2018; Zhou et al., 2019; Heriyati et al., 2020; Welch et al., 2020).

In the 1970s, Kabara’s group carried out a wide-ranging assessment of the antibacterial activities of FAs and contributed to define the modern-day field of antimicrobial lipids from a chemical viewpoint (Yoon et al., 2018). LA and GML represent the strongest antimicrobial agents in mammalian milk, they are also found in other natural sources such as coconut oil and are often used as nutritional supplements (Lieberman et al., 2006; Dayrit, 2015; Kim and Rhee, 2016).

Due to their amphipathic properties, MCFAs exhibit an antimicrobial activity through a membrane-lytic behavior causing increased cell permeability and cell lysis. In addition, MCFAs disrupt the electron transport chain either by binding to electron carriers or interfering with oxidative phosphorylation, which are vital processes for energy production in bacterial cells. Furthermore, MCFAs can directly inhibit membrane enzymes such as glucosyltransferase and also target other membrane-associated proteins (Yoon et al., 2018). In some *in silico* studies, LA has been proposed as a natural antibacterial agent via inhibiting the MurA enzyme, which is involved in bacterial cell wall biosynthesis (Heriyati et al., 2020). Galbraith and Miller (1973a, b) reported that the activity of LA was decreased by Mg^2+^ and Ca^2+^ ions and increased by lower pH, suggesting that the uptake of LA is modulated by physico-chemical properties of both the acid and the bacterial surface. MCFAs and monoglycerides mainly work in the micellar state. Monoglycerides form micelles at lower concentrations than MCFAs, which helps to clarify why monoglycerides are often more biologically potent than FAs (Jackman et al., 2020). Overall, the esterification of a fatty acid to its corresponding monoglyceride derivative enhances the antibacterial effect (Yoon et al., 2018).

Kumar et al. (2020) showed that LA inhibits the swarming motility of *P. mirabilis* in a dose-dependent manner and, at higher levels, acts on *Clostridium difficile* cell membranes and adhesins. LA can inhibit hemolysin expression, extracellular polysaccharides (EPS) and biofilm production through RsbA (a histidine-containing phosphotransmitter of two-component signaling system) dependent pathway.

Additionally, GML may almost completely kill the vegetative cells and spores of aerobic and anaerobic bacteria (Schlievert et al., 2018; Yang et al., 2018). GML also inhibits the production of staphylococcal toxic shock toxin-1 effectively and the expression of virulence factors including protein A, alpha-hemolysin, β-lactamase, and the induction of vancomycin resistance in *Enterococcus* faecalis by interfering with signal transduction (Projan et al., 1994; Ruzin and Novick, 2000).

MCFAs and monoglycerides have been suggested as natural compounds for the control of various foodborne pathogens (Kim and Rhee, 2016; Dhakal and Aldrich, 2020). Hovorková et al. (2018) discovered that the bactericidal effect of MCFAs did not exert inhibitory effects against gut commensal bacteria.

MCFAs and their monoglycerides have emerged as promising additives for replacing in-feed antibiotics and promoting sustainable animal-food production, enhancing growth performance and animal welfare (Jackman et al., 2020). Apart from the direct effects on intestinal microbiota, MCFAs could have positive effects on gut health, modulated by the degree of esterification. MCFAs can improve the intestinal morphology and function, through their beneficial effects on crypt cell renewal (Spranghers et al., 2018) and have also an immunomodulatory activity (Zhang et al., 2016). Indeed, new evidence points out that incubation of lauric acid, also found in human sebum, enhanced the innate immune defense of human sebocytes by upregulating the gene and protein expression of β-defensin-2, one of the most represented antimicrobial peptides detected in the skin (Zhou et al., 2019). In addition, LA has even been indicated as a natural antibiotic against some dermal infections, such as acne, with no toxic effect on human sebocytes (Nakatsuji et al., 2009).

As natural molecules, fatty acids have great potential and their combination with antimicrobials could reduce multidrug-resistant bacteria (Kumar et al., 2020).

## *Hermetia Illucens* as One of the Main Sources of Antimicrobial Lipids

*H. illucens* (HI), a Diptera known as the black soldier fly (Sheppard et al., 2002) is a native of tropical, subtropical, and warm temperate zones of America. It is now widespread in tropical and warmer temperate regions between about 45°N and 40°S (Makkar et al., 2014). HI has been proposed since the 1990s as an efficient way to dispose of organic waste by converting it into a protein-rich and fat-rich biomass suitable for various purposes, including animal feeding, biodiesel, oil, and chitin production (van Huis et al., 2013).

For nutritional purposes, insects’ fat was extracted from a limited number of species, i.e., HI, *Tenebrio molitor*, *Zophobas morio*, and *Bombyx mori*. Among these, HI has the highest amount of LA (up to 60%) (Spranghers et al., 2017), while in *T. molitor* it is less than 0.5% (van Huis et al., 2013; Gasco et al., 2019). HI oil also consists of various monoglycerides, diglycerides, and triglyceride, showing a very similar fatty acid profile and quality compared to that of coconut and palm kernel oil (Ushakova et al., 2016; Muller et al., 2017; Spranghers et al., 2017). LA concentration and synthesis may, however, undergo small variations according to the substrate used, to the shift from lipogenesis to glycogenesis related to the development stage, to the extraction methods, and the killing method and storage (Alifian et al., 2019; Caligiani et al., 2019; Rabani et al., 2019; Ewald et al., 2020; Kierończyk et al., 2020).

The high fat content of the prepupae could limit their use as a feed ingredient. Thus, it could be interesting to partially extract the fat from the prepupal meal and use a sufficient amount of LA in the feed adding value to it, while the extracted part could be suitable for other purposes, such as the production of biofuel (Spranghers et al., 2017).

To reduce the costs for lipid extraction, the major challenges are large-scale production and legal frameworks to allow the use of insects as ingredients for food and feed. So far, insect lipids are allowed in feeding all animal species, but PAPs (processed animal proteins) are only permitted in aquaculture and the possibility of extending the authorization of their use to poultry and swine feed is still pending (Sogari et al., 2019).

HI is not considered a disease vector, since the adult fly is not attracted to human habitats or foods and the eggs are never laid on decaying organic material. Furthermore, the prepupae process organic waste very quickly and empty their digestive tract, limiting bacterial proliferation (van Huis et al., 2013; Makkar et al., 2014; Muller et al., 2017). Moreover, the larvae modify the microflora of manure, potentially reducing harmful bacteria such as *Escherichia coli* 0157:H7 and *Salmonella enterica* (Makkar et al., 2014).

HI is characterized by an immune system in which cell-mediated and humoral innate mechanisms work jointly. Hemolymph cells are involved in cellular immune responses, while phenoloxidase, antimicrobial peptides (AMPs), and proteins belong to humoral innate response (Zdybicka-Barabas et al., 2017). HI contains chitin which exhibits antimicrobial properties (Borrelli et al., 2017).

Up until recently, research has examined different strategies to take advantage of HI immunity. As reported, the modulation of the substrate where the larvae are fed could induce the expression of different proteins and specific immunity gene proteins with a different spectrum of antimicrobial activity (Zdybicka-Barabas et al., 2017; Vogel et al., 2018).

## Antimicrobial Properties of *Hermetia Illucens* Larvae Inclusion in Animal Feeding

As natural and edible antimicrobial products, MCFAs have been proposed as alternatives to conventional antibiotic growth promoters in livestock nutrition (Fortuoso et al., 2019; Zhou et al., 2019). In the last few years, the use of insect-based diet as HIL meal and oil in substitution of the conventional feedstuffs has been investigated showing promising findings in terms of nutritive value and low environmental impact, with negligible effects on lipid digestibility, performance parameters, and animal health in the swine, poultry and rabbit industry (Cullere et al., 2016, 2018, 2019a; Dalle Zotte et al., 2018, 2019; Secci et al., 2018; Gariglio et al., 2019; Gasco et al., 2019; Yu et al., 2019, 2020).

To date, data on the antimicrobial effects of insect-based diet *in vivo* are scarce. All available studies are summarized in Table 1.

**TABLE 1:** Antimicrobial effect of insect-derived LA and GML in *in vivo* animal studies.

Elicitors	Animal species	Key findings	References
HI meal	Weaned piglets	2 log fold reduction of D-Streptococci	Spranghers et al., 2018
HI meal	Weanling piglets	Increased number of *Lactobacillus* and *Bifidobacterium*, quadratically decreasing number of *E. coli*	Yu et al., 2020
HI meal	Finishing pigs	Decreased abundance of *Streptococcus* spp., increased number of *Lactobacillus*, higher concentrations of total SCFA, upregulation of anti-inflammatory cytokine (IL-10)	Yu et al., 2019
HI oil	Growing rabbits	Significantly lower growth of *Yersinia enterocolitica*, positive influence on the cecal microbiota	Dabbou et al., 2020
HI meal	Siberian sturgeon	Positive effect on the gut microbiota composition and intestinal morphology	Józefiak et al., 2019
HI oil	Young turkey	Reduced growth of Enterobacteriaceae, decreasing levels of IL-6	Sypniewski et al., 2020

## Insect-Based Food Perspectives in Human Nutrition

The nutritional values of available insects differ according to species, effects of diet or substrate, and environmental conditions. However, it is interesting that the value of some insect species in insect products is better than meat. The risk of consuming edible insects for humans compared with consuming other animal products or food protein sources is lesser-known. The EFSA has highlighted a lack of data regarding microbiology, virology, parasitology, and toxicology of edible insects. Currently, there is very limited information on the risks associated with families or species of insects, details of the manufacturing processes and the environmental impact of different farming systems, and there is also a lack of human consumption data (Ferri et al., 2019; van Huis, 2020).

Over recent decades, human nutrition has undergone dramatic changes such as an increased intake of partially hydrogenated oils and trans fatty acids, which may lead to a pro-inflammatory state, associated with obesity, type 2 diabetes, and other epidemic metabolic disorders in western countries (van den Brink et al., 2019).

The insect bioactive peptides and lipids have beneficial effects on human health, such as antioxidant, antimicrobial and antidiabetic properties, angiotensin I converting enzyme (ACE) inhibition activity, effects against inflammation and cancer. Insects can be also used as functional food ingredients (Dutta et al., 2019; da Silva Lucas et al., 2020).

Insect diet might also promote metabolic shifts, including the increased production of microbiota-derived short-chain fatty acids (SCFAs) such as butyrate, acetate, and propionate (Borrelli et al., 2017), exhibiting antibacterial activity against various pathogens (Yang et al., 2018). SCFAs improve mucosal and systemic innate and acquired immune responses to control inflammation during infections and reinforce homeostasis. Butyrate also inhibits histone deacetylase 3 to confer macrophages with non-inflammatory enhanced antimicrobial activity (Shinde et al., 2020).

As possible drawbacks for human nutrition, insect lipid fraction, unlike vegetable oils, is low in poly-unsaturated Fatty Acids (PUFA). As new alternatives to improve insect fats, the modulation of the substrate for the larvae by using fish-offal waste could increase the *n*− 3 proportion in *Hermetia illucens* larvae meal and oil, as well as the direct inclusion of a PUFA-rich feed ingredient (i.e., linseed) in animal diets, could be a useful strategy to provide healthier meat for human consumption (Cullere et al., 2019b; Caimi et al., 2020). Moreover, MCFAs show low solubility and unpleasant odors that stimulate the release of cholecystokinin and reduce the feed intake of animals. Encapsulation of MCFAs could be a suitable solution to overcome these limits (Zhou et al., 2019).

Due to their nutrient profile, insects could also be a game changer in the race to fight hunger, food insecurity, malnutrition (da Silva Lucas et al., 2020). There are many articles on the antimicrobial effects of GML *in vitro* and *in vivo* in animal studies but only three papers highlight *in vivo* antimicrobial effects in humans (Barker et al., 2019). However, other *in vivo* human studies and further research is recommended.

## Concluding Remarks

Antimicrobial resistance (AMR) is an impending public health crisis. As we respond to the COVID-19 pandemic, we are seeing what our health systems look like, with limited treatments available to tackle an outbreak. To stem the rise of AMR infections, physicians, veterinarians, and environmental scientists must all remain vigilant and maintain a one health view. This mini review shows how *Hermetia illucens* (HI) represent a good candidate source of LA with a potential role in the AMR scenario. In particular, HI-derived oil might be useful in protecting against microbial infections, modulating inflammation, healing wounds, and controlling the balance and distribution of bacteria in gut microbiota. The presence of insect-derived LA in animal feed could prevent the use of conventional antibiotics in meat production, limiting the diffusion of microbes harboring AMR genes (Dabbou et al., 2020).

If whole insects are used as feed, the antimicrobial molecules must remain stable during feed processing and digestion. Thus, these compounds could be purified and identified directly from larval extracts, or it would be possible to characterize *in vitro* HI immunity-related genes with the aim to discover novel antimicrobials and molecules (De Smet et al., 2018).

The MCFAs composition of HIL oil is very similar and can replace palm kernel or coconut oil, as additives and even pharmaceuticals (Matthäus et al., 2019; Rabani et al., 2019). Both vegetable oils are products of plants from tropical climates, where they are abundant. The impact on the environment is also considerable, as an increase in demand for vegetable oils and biofuels contributes to water waste, tropical deforestation, loss in biodiversity, and habitat fragmentation. For this reason, palm kernel and coconut oil are currently criticized from an ecological point of view (Wang et al., 2017; Matthäus et al., 2019; Verheyen et al., 2020; Kierończyk et al., 2020). Therefore, in a vision of the circular economy, it would be possible to manage, reduce, and efficiently use organic waste for HIL rearing as a sustainable source of nutrients (according to the 4R of the EU Parliament Directive no. 2008/98 and the other Directive 94/62/EC).

The Hippocratic concept that “*we are what we eat*” is present in all cultures and it represents one of the biggest barriers to the consumption of insects in food neofobic western societies, which consider entomophagy to be disgusting (van Huis et al., 2013). The scientific community and all stakeholders must now make efforts to promote insects as a source of bioactive peptides and lipids and to prevent the next global threat of antimicrobial resistance as well as metabolic disorders.

If “we are what we eat,” but obesity, diabetes, cancer, and antimicrobial resistance are constantly increasing in western society, why not eat insects?

## Author Contributions

LB and LV conducted the literature searches, conceptualized, and wrote the manuscript. LD and AP critically revised the manuscript. LM and AF read, revised, and concurred with the final version of the review. All authors have made an intellectual contribution to the work and approved the submitted version.

## Conflict of Interest

The authors declare that the research was conducted in the absence of any commercial or financial relationships that could be construed as a potential conflict of interest.
